# Cross-linking/mass spectrometry at the crossroads

**DOI:** 10.1007/s00216-020-02700-x

**Published:** 2020-05-29

**Authors:** Lolita Piersimoni, Andrea Sinz

**Affiliations:** Department of Pharmaceutical Chemistry & Bioanalytics, Institute of Pharmacy, Charles Tanford Center, Martin Luther University Halle-Wittenberg, Kurt-Mothes-Str. 3a, 06120 Halle (Saale), Germany

**Keywords:** Chemical cross-linking, Cleavable cross-linkers, Electron microscopy, Mass spectrometry, Protein conformation, Protein-protein interactions, Structural biology

## Abstract

Cross-linking/mass spectrometry (XL-MS) has come a long way. Originally, XL-MS was used to study relatively small, purified proteins. Meanwhile, it is employed to investigate protein-protein interactions on a proteome-wide level, giving snapshots of cellular processes. Currently, XL-MS is at the intersection of a multitude of workflows and the impact this technique has in addressing specific biological questions is steadily growing. This article is intended to give a bird’s-eye view of the current status of XL-MS, the benefits of using MS-cleavable cross-linkers, and the challenges posed in the future development of this powerful technology. We also illustrate how XL-MS can deliver valuable structural insights into protein complexes when used in combination with other structural techniques, such as electron microscopy.

**Figure:**
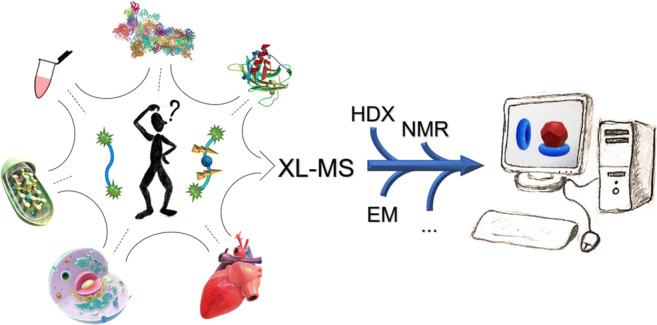
Graphical abstract

Graphical abstract

## Cross-linking/mass spectrometry: current status and future challenges

**Fig. 1:**
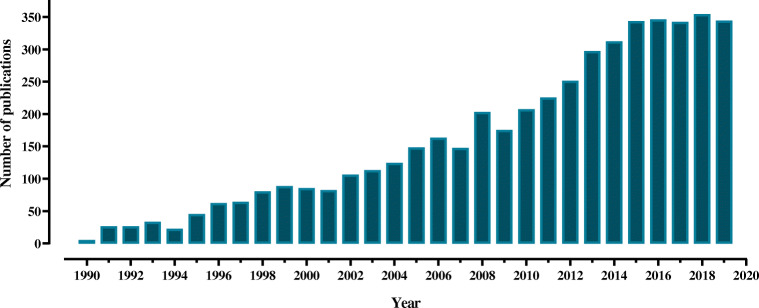
Annual number of publications from 1990 to 2019, as listed in the Web of Science for the search terms “cross-linking mass spectrometry” or “crosslinking mass spectrometry”

Cross-linking/mass spectrometry (XL-MS) has undergone an impressive transformation during the last two decades, developing from a niche technique to a generally accepted method for protein structure analysis. Since 2015, the number of annual publications for XL-MS has been leveling at ca. 350 (Fig. 1). Continuous improvements in cross-linking reagents [1–3], MS instrumentation [4], and software tools [5], however, have laid the foundation for the recent inroads the XL-MS approach is taking to address important questions in structural biology.

**Fig. 2:**
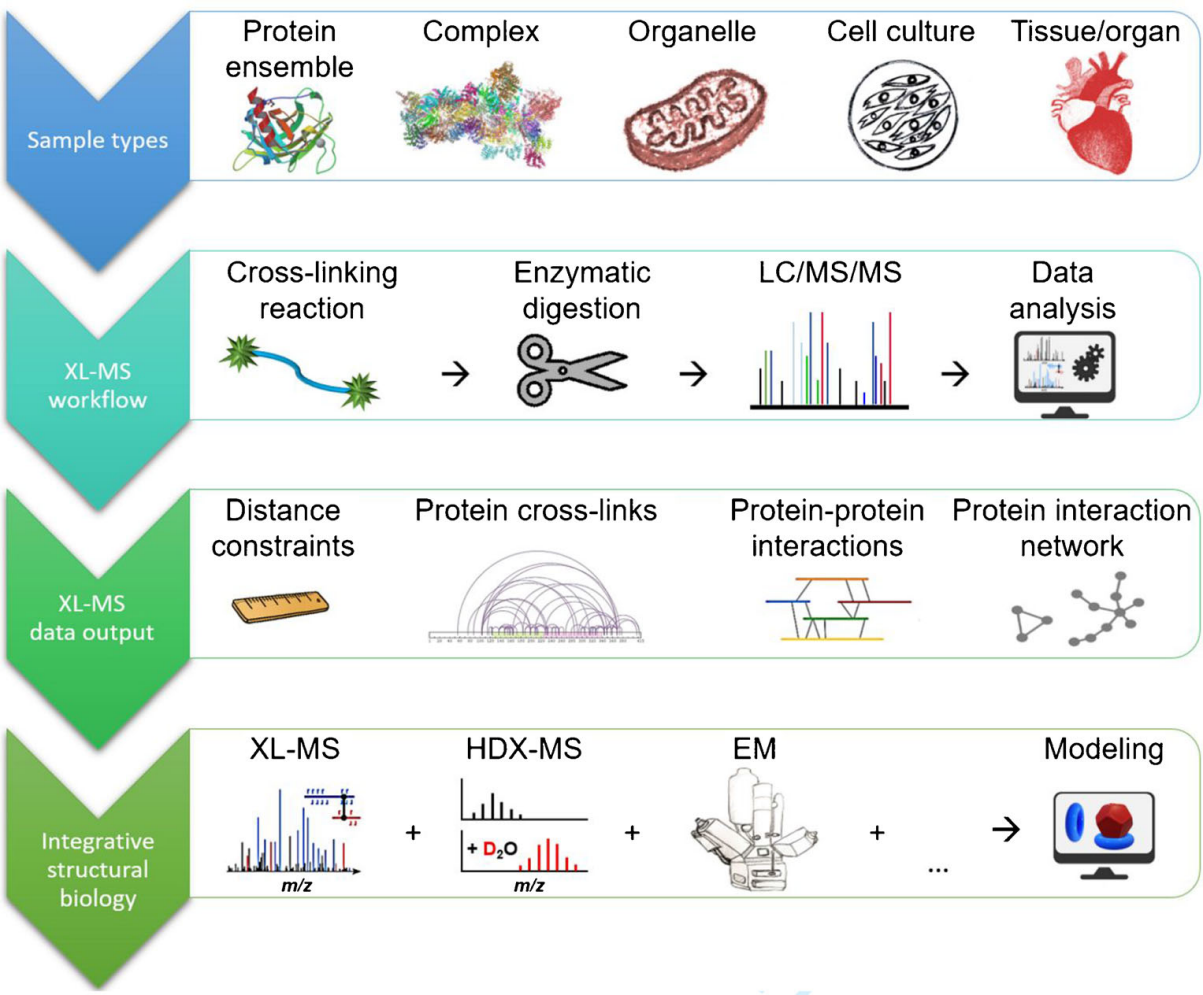
XL-MS workflows allow the identification of cross-links in purified proteins, cell culture, and intact tissue. XL-MS yields structural information in the form of distance constraints on single proteins, protein assemblies, and protein interaction networks. Moreover, it serves as an integrative method in structural biology when combined with other structural techniques, like hydrogen/deuterium exchange (HDX)-MS or electron microscopy (EM)

XL-MS is now being increasingly used to determine protein-protein interaction networks at the system-wide level, delivering unprecedented insights compared to other methods, while at the same time requiring minimal amounts of sample. Consequently, XL-MS has expanded from studying purified proteins and their assemblies towards investigating living cells, tissues, and even whole organisms (Fig. 2).

To deal with the enormous complexity of samples in proteome-wide network studies, a number of methodological challenges have to be overcome [6–9]. Currently, the maximum number of cross-links identified in system-wide studies is approximately 10,000 and it remains to be seen how far this limit can be pushed. One of the greatest challenges when conducting proteome-wide XL-MS experiments is the bias towards high-abundance proteins. A recent study addressed this issue by using a crowded cellular environment mimicked in vitro and eukaryotic cell lysates to increase the detection of low-abundance proteins on a proteome-wide scale [10]. This emphasizes the importance of developing XL-MS protocols reflecting system-wide protein-protein interactions in a comprehensive, near-in situ manner.

The great variety of XL-MS workflows currently available makes it challenging for newcomers, as well as for experts, to choose the optimum protocol for a specific biological question. This variety in workflows is reflected by a community-wide XL-MS study conducted recently by 32 groups participating worldwide [11].

The first question to address is which cross-linker to choose. Clearly, amine-reactive *N*-hydroxysuccinimide (NHS) esters targeting lysine residues, but also serines, threonines, and tyrosines, are the cross-linkers overwhelmingly used to date. As far as the nature of the cross-linker itself is concerned, i.e., whether cleavable versus non-cleavable, approximately 78% of all studies currently make use of non-cleavable cross-linkers, such as disuccinimidylsuberate (DSS) and bis(sulfosuccinimidyl)suberate (BS^3^) [12]. Both cross-linkers differ only in a sulfonic acid group that is incorporated into BS^3^ to enhance water solubility and to bridge a distance of 11.4 Å, resulting in Cα-Cα distances of ~ 27 Å. MS-cleavable cross-linkers, such as disuccinimidyl sulfoxide (DSSO) and disuccinimidyldibutyric urea (DSBU) (Table 1), are increasingly being used as they allow targeted identification of the cross-linked product based on characteristic fragments generated during MS/MS experiments. The main spacer lengths of cross-linkers range between 10 and 12.5 Å, based on the most frequently used cross-linkers, BS^3^ and DSS (both non-cleavable), as well as DSSO and DSBU (both MS-cleavable), used in the community-wide XL-MS study [11].

**Table 1:**
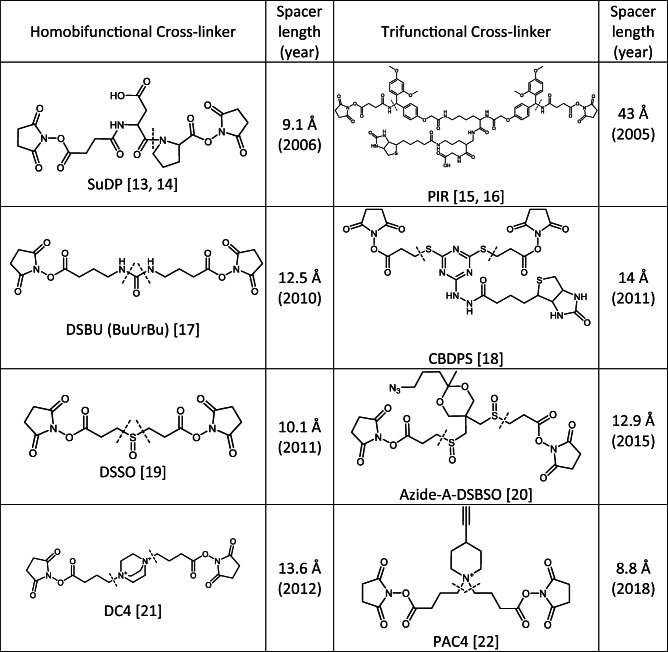
Structures and spacer lengths of selected MS-cleavable cross-linkers; dashed lines indicate cleavage sites

## Are MS-cleavable cross-linkers the key to success in XL-MS?

**Fig. 3:**
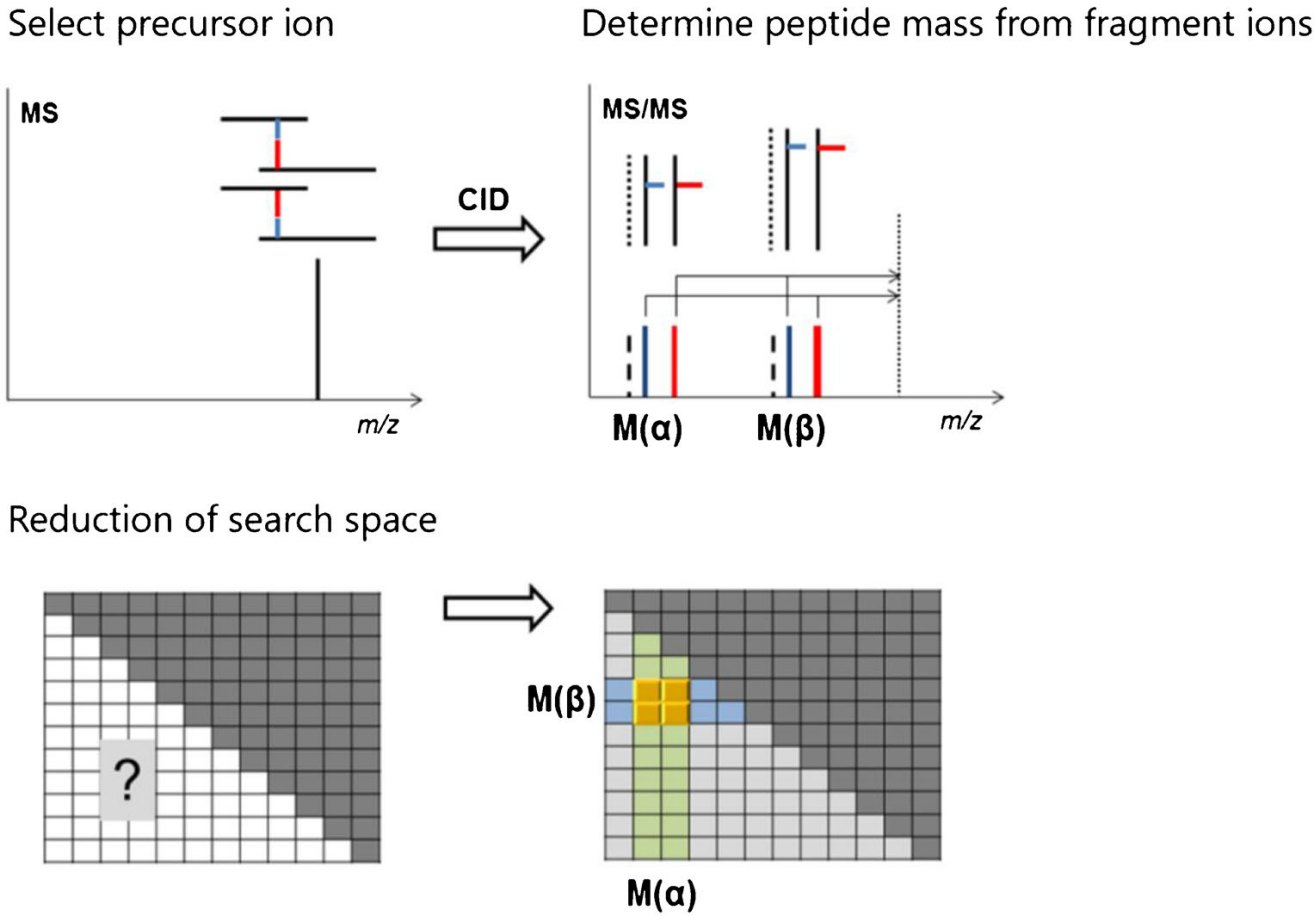
Reduction of search space for MS-cleavable cross-linkers. After MS selection of a cross-linked product as a precursor ion, the masses of the connected peptides can be inferred from the fragment ions created in MS/MS experiments

As outlined above, more than three-quarters of XL-MS studies are currently still performed with non-cleavable cross-linkers. The reason for this is that non-cleavable cross-linkers, like BS^3^ and DSS, are used in many structural biology labs in well-established and tested protocols for studying proteins and relatively small protein assemblies. However, as the field is now increasingly moving towards conducting proteome-wide XL-MS studies, MS-cleavable cross-linkers will be crucial to make handling of these highly complex reaction mixtures easier and faster. The distinct advantage of MS-cleavable cross-linkers is that they release characteristic fragment ions during collision-induced dissociation (CID)-MS/MS experiments in the mass spectrometer [23]. As only the linearized peptides are identified, the quadratic search space (*n*^2^ problem) is reduced to a linear search space (2*n* problem) (Fig. 3) [24]. This allows an unambiguous assignment of cross-links, even in highly complex mixtures, such as living cells, tissues, or whole organisms.

To generate the characteristic fragment ions in MS/MS experiments, a variety of MS-cleavable cross-linkers are available. All currently available MS-cleavable reagents contain labile bonds as cleavage sites in their spacer chains that are usually fragmented by CID-MS/MS (Table 1). MS-cleavable reagents may additionally contain a third group for an affinity-based purification of the cross-linked products, such as biotin, to enrich the cross-linked products and increase the number of identified cross-links. Alternatively, the affinity handle can be introduced after the cross-linking reaction, for example, by utilizing a “click” reaction between an alkyne and an azide. This offers the distinct advantage of ensuring that the protein interfaces are not to be disturbed by a bulky biotin label. To address the interactions of complete proteomes in a comprehensive manner, more cross-linkers will be needed in the future, which are MS-cleavable and can be enriched.

In 2005, the class of protein interaction reporter (PIR) cross-linkers was presented, comprising reagents that are MS-cleavable and can be enriched via a biotin label [15, 16]. A MS-cleavable cross-linker introduced in 2006 is the SuDP linker, which contains a labile aspartate-proline bond [13]. A cross-link identification strategy was presented for SuDP [14] that, in principle, formed the basis for the subsequent development of proteome-wide XL-MS workflows using other MS-cleavable linkers. Between 2010 and 2012, the MS-cleavable linkers DSBU [17], DSSO [19], CDBPS [18], and DC4 [21] were introduced and applied in various protein systems. Derived from DSSO, two trifunctional cross-linkers were designed harboring azide (azide-A-DSBSO) or alkyne (alkyne-A-DSBSO) groups to enable affinity purification strategies based on click chemistry [20]. Similarly, the trifunctional PAC4 linker was developed from DC4 containing an alkyne group for an affinity enrichment of cross-links [22]. An exciting and novel approach for the enrichment of cross-linked products is provided by the PhoX linker [25]. PhoX is non-cleavable but contains a phosphonic acid group, therefore allowing cross-linked products to be enriched by routine strategies for phosphopeptide analysis, such as immobilized metal ion affinity chromatography (IMAC).

In the next section, we will illustrate how the MS-cleavable cross-linkers can be used in combination with complementary structural methods to gain insights into the structure of protein complexes when high-resolution techniques fail.

## Integration of XL-MS with complementary structural methods to address biological questions

The increasing popularity of XL-MS (Fig. 1) is largely attributed to its strength as an integrative method in structural biology, aiming to create more confident models of target proteins. A number of different techniques can be used in combination with XL-MS, such as HDX-MS, EM, X-ray crystallography, nuclear magnetic resonance spectroscopy, and small-angle X-ray scattering. There are numerous programs designed to combine the structural data obtained from these techniques, and these help in determining the dynamics of protein assemblies, such as the Integrative Modeling Platform [26] and HADDOCK [27].

**Fig. 4:**
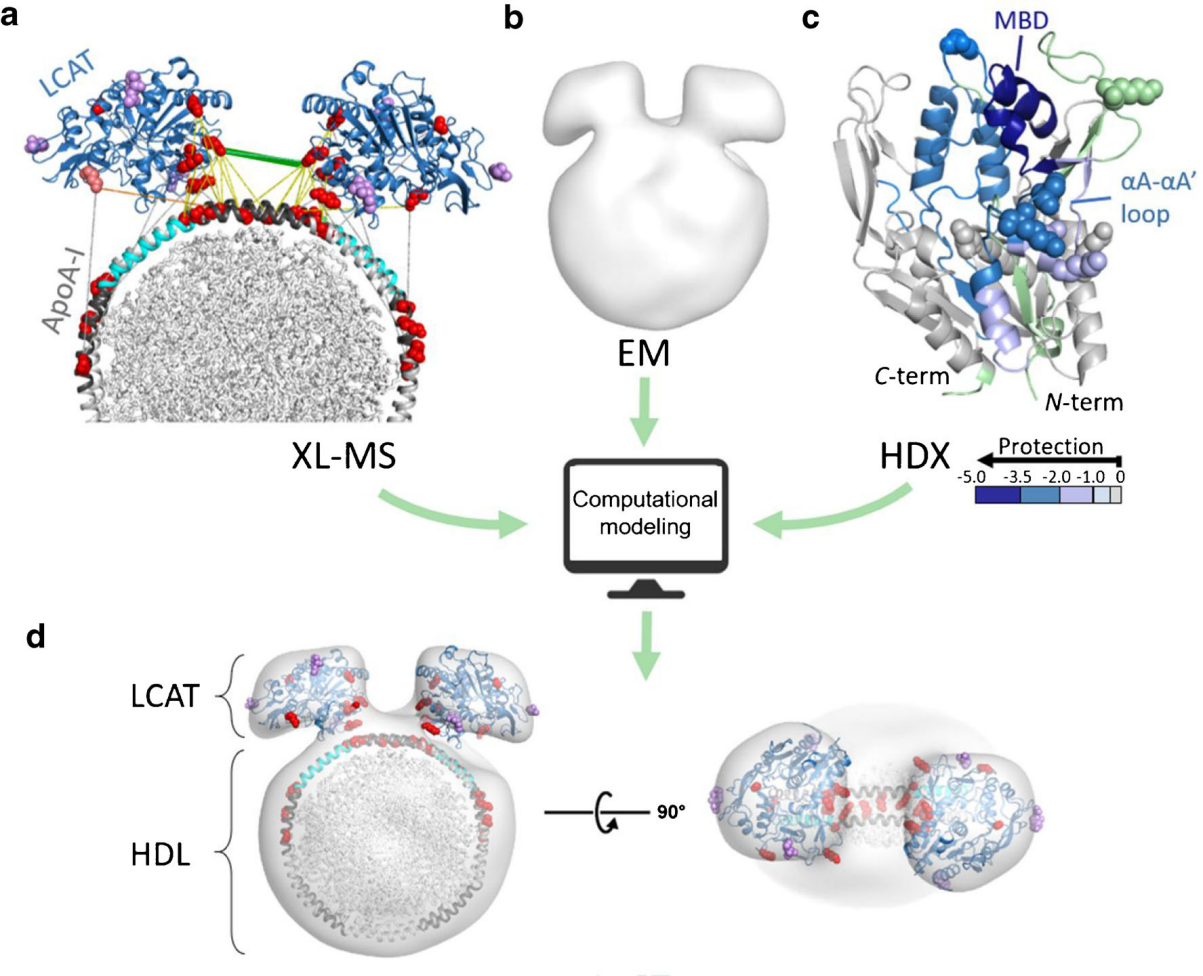
Integration of XL-MS with the complimentary structural methods of EM and HDX-MS data for the LCAT-HDL complex. **a** Representation of the inter-protein cross-links. Red spheres indicate reactive amino acids; cross-links within the distance limit (Cα-Cα distance < 40 Å) are shown in yellow, the specific cross-link connecting two LCAT molecules is shown in green, ApoA-I α-helices 6 are presented in cyan, and α-helices 4 and 5 are shown in dark gray. **b** EM 3D density map. **c** HDX-MS data presented in the LCAT structure (PDB 6MVD); LCAT regions with decreased deuterium exchange after HDL binding are presented on a scale of blue intensity. Spheres present amino acids involved in the cross-links with ApoA-I. No HDX data were obtained for the regions shown in light green. **d** Proposed structural model of the LCAT-HDL complex obtained by integrating all experimental data. The figure was adapted from [28]

A recent study presents an excellent example in which XL-MS was employed in combination with HDX-MS and negative-stain EM to refine the molecular details of the lecithin:cholesterol acyltransferase (LCAT) interaction with high-density lipoprotein (HDL), in particular with its prominent component apolipoprotein A-I (ApoA-I) [28]. High-resolution structural analysis of the HDL-LCAT complex has been hampered so far by its highly dynamic and heterogeneous nature, making it an ideal target for investigation by a combination of low- and medium-resolution techniques. First, a low-resolution 3D density map of the LCAT-HDL complex was reconstructed from negative-stain EM images, revealing that LCAT binds at the edge of the HDL, which accomodates two ApoA-1 molecules in a belt-like arrangement (Fig. 4). This is in good agreement with previous biochemical findings showing that ApoA-I acts as a direct activator of LCAT [29]. ApoA-I contains ten tandem amphipathic α-helices following an *N*-terminal globular domain. Two ApoA-I molecules were found to be arranged in an antiparallel manner and aligned at their central α-helix 5 [28], which is in agreement with the discoid model [30]. The envelope of the LCAT-HDL complex was reconstituted from EM images and guided further modeling and docking studies by integrating XL-MS and HDX-MS data, while also taking previous biochemical and structural knowledge into account [29–32]. For XL-MS, the complex reconstituted in vitro was cross-linked with the MS-cleavable DC4 linker (Table 1) [21], enabling an unambiguous identification of cross-linked peptides based on the characteristic fragment ions in MS/MS spectra. DC4 possesses a spacer length of 13.6 Å and was therefore assumed to bridge lysines with Cα-Cα distances of up to 40 Å. XL-MS data clearly indicated the presence of distinct “hotspots” in α-helix 5 of ApoA-I, as well as at the boundaries with the two adjacent α-helices 4 and 6 (Fig. 4a). These hotspots were consistent with α-helix 6 being the preferred binding site for LCAT [29]. They were also in-line with a previous HDX-MS study, suggesting an increased protection in a region adjacent to α-helix 6 in the presence of LCAT [32]. As reported in [28], HDX-MS experiments were performed with LCAT in the absence and presence of HDL showing a decrease of deuterium exchange in the membrane-binding domain and in the hydrophobic αA-αA′ loop (Fig. 4c) upon complex formation. These protected regions of LCAT gave strong hints how the complex was stabilized by direct interactions with HDL, which was further supported by the observation of cross-links in the same regions, as well as in regions adjacent to the membrane-binding domain. As these sites are located in the same hydrophobic regions of LCAT in the X-ray structures [31, 32], this gave further support to the interpretation that these LCAT sites interact with HDL.

The EM 3D density map of the LCAT-HDL complex showed two LCAT molecules in close spatial proximity on HDL (Fig. 4b). XL-MS supported this overall orientation of two LCAT molecules via one specific cross-link connecting the same amino acid of two LCAT molecules (Fig. 4a). This cross-link also defined the C2 symmetry of the LCAT orientation in the two lobes of the EM density map. It should be noted that the proposed model was not the only solution for the LCAT-HDL complex. The structural data obtained could not be satisfied with a single docking model, suggesting that this complex is a heterogeneous and dynamic system. Due to the presence of two main LCAT-HDL complex populations containing one or two molecules of LCAT that additionally exhibit different angular spacings between the two LCAT molecules, some of the cross-links were found to exceed the Cα-Cα distance limit of 40 Å.

This study exemplifies how different low-resolution methods can be combined to overcome the inherent limitations of each single technique. If complementary results are integrated and computationally organized, more accurate structural models can be generated for proteins and protein complexes, especially for those that are heterogeneous and challenging to be studied by conventional high-resolution structural techniques.

## Outlook

XL-MS has impressively revealed its power for investigating purified proteins as well as protein interaction networks, as is illustrated by the large body of literature that has accumulated during the past 20 years. Undoubtedly, XL-MS will have a bright future as an integrative method in structural biology when used in combination with other methods, most importantly probably cryo-EM. A number of different workflows have been developed recently in different labs, indicating that MS-cleavable cross-linkers could offer great benefits for system-wide XL-MS. On the other hand, workflows with non-cleavable cross-linkers are still employed in the majority of XL-MS studies for the investigation of purified proteins or pre-fractionated protein samples. It can be envisioned that two main general XL-MS workflows will prevail in the next 5 years, one for purified proteins and relatively small protein assemblies (using non-cleavable cross-linkers), one for system-wide XL-MS studies (using cleavable cross-linkers). Currently, the most urgent task in the XL-MS community is to harmonize protocols and data reporting formats to ensure obtaining reproducible and reliable results. A recently conducted, community-wide XL-MS harmonization study revealed a great variety in the protocols employed as well as in the outcome, i.e., the number of identified cross-links, between the 32 participating groups [11]. This underlines the need for establishing generally accepted XL-MS protocols as well as common formats for data analysis and reporting results.

## Abbreviations

ApoA-I: Apolipoprotein A-I
Azide-A-DSBSO: Azide*-*tagged, acid-cleavable disuccinimidyl bissulfoxide
BS^3^: Bis(sulfosuccinimidyl)suberate
BuUrBu: 4-{3-[3-(2,5-Dioxo-pyrrolidine-1-yloxycarbonyl)-propyl]-ureido}-butyric acid 2,5-dioxo-pyrrolidine-1-yl ester
CBDPS: Cyanurbiotindipropionyl succinimide
CID: Collision-induced dissociation
DC4: 1,4-Bis{4-[(2,5-dioxo-1-pyrrolidinyl)oxy]-4-oxobutyl}-1,4-diazoniabicyclo[2.2.2]octane
DSBU: Disuccinimidyldibutyric urea
DSS: Disuccinimidylsuberate
DSSO: Disuccinimidyl sulfoxide
EM: Electron microscopy
HDL: High-density lipoprotein
HDX: Hydrogen/deuterium exchange
IMAC: Immobilized metal ion affinity chromatography
LCAT: Lecithin:cholesterol acyltransferase
MS: Mass spectrometry
MS/MS: Tandem mass spectrometry
NHS: *N*-Hydroxysuccinimide
PAC4: 1,1-Bis{4-[(2,5-dioxopyrrolidin-1-yl)oxy]-4-oxobutyl}-4-ethynylpiperidin-1-ium
PIR: Protein interaction reporter
SuDP: Disuccinimidylsuccinamyl aspartyl proline
XL-MS: Cross-linking/mass spectrometry
